# Genomic and Transcriptomic Characterization of Umatilla Virus Isolated and Identified from Mosquitoes in Ningxia, China

**DOI:** 10.3390/microorganisms13122717

**Published:** 2025-11-28

**Authors:** Kun Han, Yuhong Yang, Long Wang, Liqin Yu, Ruichen Wang, Xiaoyu Gu, Fan Li, Qikai Yin, Shihong Fu, Kai Nie, Qianqian Cui, Songtao Xu, Huanyu Wang

**Affiliations:** 1 Ningxia Center for Disease Control and Prevention, Yinchuan 750011, China; khan2006@163.com (K.H.); nxwl76@163.com (L.W.); 2 National Key Laboratory of Intelligent Tracking and Forecasting for Infectious Diseases, NHC Key Laboratory of Biosafety, National Institute for Viral Disease Control and Prevention, Chinese Center for Disease Control and Prevention, Beijing 102206, China; yyh19980804@163.com (Y.Y.); yulq981029@163.com (L.Y.); wangrc96@163.com (R.W.); guxiaoyu905@163.com (X.G.); lifan@ivdc.chinacdc.cn (F.L.); yinqk@ivdc.chinacdc.cn (Q.Y.); fush@ivdc.chinacdc.cn (S.F.); niekai@ivdc.chinacdc.cn (K.N.); cuiqq@ivdc.chinacdc.cn (Q.C.); xust@ivdc.chinacdc.cn (S.X.)

**Keywords:** Umatilla virus, *Orbivirus*, *Culex pipiens pallens*, Ningxia Hui Autonomous Region, genomic characterization, transcriptomic characterization

## Abstract

During the 2023 surveillance of mosquito-borne viruses in Ningxia Hui Autonomous Region, a strain of Umatilla virus (UMAV) was isolated from a pool of *Culex pipiens pallens* (NX23166) collected in Xiji County and cultured in C6/36 cells. Electron microscopy revealed that NX23166-infected mosquito cells showed approximately 70-nm virus particles, typical of the genus *Orbivirus*. Through next-generation sequencing, 10 double-stranded RNA (dsRNA) segments of the virus were obtained. Phylogenetic and homology analyses based on these sequences revealed that this strain was most closely related to the first Chinese isolate from Yunnan in 2013 (DH13M98) and an Australian isolate from 2015 (M4941_15). However, the VP3 protein of this strain showed the closest evolutionary relationship to a German isolate from 2019 (ED-I-205-19), with an amino acid sequence identity of 94.00%. In contrast, the identity of the VP3 protein to that of other strains ranged only from 47.38% to 51.49%, suggesting that these two strains may belong to the same serotype. Nevertheless, this hypothesis needs to be further verified by a serum neutralization test. Furthermore, transcriptome sequencing analysis showed that infection with the Ningxia isolate of UMAV induced significant temporal transcriptomic reprogramming in C6/36 cells. This reprogramming was characterized by early activation of innate immune responses such as the Toll signaling pathway and autophagy, followed by significant suppression of metabolic pathways, including oxidative phosphorylation in the mid to late stages of infection, demonstrating a molecular phenotype of coordinated immune activation and metabolic suppression. These results provide new insights into the genetic diversity and geographic distribution of the species UMAV.

## 1. Introduction

The genus *Orbivirus*, which has the highest number of species among the seven genera in the family *Sedoreoviridae*, currently includes 22 virus species recognized by the International Committee on Taxonomy of Viruses (ICTV) (https://ictv.global/taxonomy; accessed on 18 August 2025). Viruses within this genus have a wide host range, including humans, livestock, wild ruminants, equines, marsupials, sloths, bats, and birds, as well as hematophagous arthropods (ticks, midges, and mosquitoes) [1,2,3]. Certain orbiviruses are significant animal pathogens, such as bluetongue virus (BTV), African horse sickness virus (AHSV), and epizootic hemorrhagic disease virus (EHDV). These pathogens can cause severe hemorrhagic diseases in sheep, cattle, horses, deer, and other ungulates, posing a serious threat to the livestock industry [4,5,6].

Viruses in the genus *Orbivirus* are non-enveloped and have icosahedral symmetry. Their genomes consist of 10 double-stranded RNA (dsRNA) segments, which are designated segment 1 to segment 10 in order of decreasing size. These segments encode seven distinct structural proteins (VP1-VP7) and at least three or four distinct nonstructural proteins (NS1, NS2, NS3/NS3a, and NS4) [7,8]. The structural RNA-dependent RNA polymerase viral protein (VP1) is highly conserved among orbiviruses [9]. In contrast, the outer-capsid protein one (OCP1), which determines the serotype, is highly variable in both its amino acid sequence and size [3]. OCP1 is encoded by segment 2 (VP2) in Culicoides/Sand fly-borne orbiviruses (CBOs/SBOs), by segment 3 (VP3) in mosquito-borne orbiviruses (MBOs) such as the Umatilla virus (UMAV), and by segment 4 (VP4) in tick-borne orbiviruses (TBOs) [10,11,12,13].

The species *Umatilla virus* comprises 5 serotypes: *Umatilla virus*, *Minnal virus* (MINV), *Netivot virus* (NETV), *Llano Seco virus* (LLSV), and the new member *Stretch Lagoon orbivirus* (SLOV) [14]. UMAV was first isolated from *Culex pipiens* in Umatilla County, Oregon, USA, on 30 July 1969. The virus was later isolated from a sparrow (*Passer domesticus*) sample collected in Texas, USA, in 1967 [11]. It has also been isolated in *Culex tritaeniorhynchus* mosquitoes collected in Mangshi City, Yunnan Province, China, in 2013 [3]. In 2019, UMAV was detected in the liver tissue from a Cape penguin (*Spheniscus demersus*) that died of hepatitis at a zoo in Germany [15]. The other three established serotypes (LLSV, MINV, and NETV) were isolated from mosquitoes collected in the United States, India, and Israel, respectively [3]. SLOV was isolated from *Culex annulirostris* collected in the Kimberley region of Western Australia in 2002 [16]. Koyama Hill virus (KHV) was isolated from *Culex sasai* collected in Japan in 2011 [17]. Sequence analysis showed that both SLOV and KHV belong to UMAV.

This study reports the first isolation and characterization of a UMAV strain (NX23166) from the Ningxia region of northern China. Genomic characterization and transcriptomic analysis were conducted, which not only enriches the viral resource library in China but also provides new insights into the genetic diversity and geographical distribution of this virus.

## 2. Materials and Methods

### 2.1. Samples and Cells

A total of 5540 mosquito samples were collected from livestock pens in Helan County, Dawukou District, Xiji County, and Pingluo County of the Ningxia Hui Autonomous Region between July and September 2023. These samples were divided into 185 groups based on collection location and mosquito species. Mosquitoes were identified morphologically with reference to *Classification and Identification of Important Medical Insects of China* [18]. In a previous study, we had screened all mosquito samples for six important mosquito-borne viruses (Tahyna virus, Japanese encephalitis virus, Sindbis virus, West Nile virus, Culex flavivirus, and Getah virus) [19]. Building on this work, the current study further conducted virus isolation on all mosquito samples. Subsequently, they were pooled into groups of 30 mosquitoes per tube based on species and location. All samples were transported in liquid nitrogen and stored at ultra-low temperatures until use. All cells used in this study were obtained from our laboratory’s cell bank. These included mosquito cell line C6/36, human cell lines SW-13 and Huh-7, mouse cell line BHK-21, canine cell line MDCK, porcine cell line PK-15, and monkey cell line Vero.

### 2.2. Virus Isolation and Cell Growth Kinetics

Mosquitoes were removed from the liquid nitrogen and immediately homogenized and centrifuged as previously reported [20]. The 70 μL supernatants were inoculated onto monolayers of C6/36 or BHK-21 cells in 24-well plates and incubated at 28 °C and 37 °C with 5% CO_2_, respectively. Cells were observed for cytopathic effects (CPEs) every 24 h for 5–7 days. A virus was considered successfully isolated if it induced CPEs in three successive cell passages. Infected cell supernatants were stored at −80 °C for further analysis.

The growth kinetics of the positive isolate was characterized using C6/36, SW-13, Huh-7, BHK-21, MDCK, PK-15, and Vero cells. Supernatants were harvested at 0, 24, 48, 72, and 96 h post-inoculation (h.p.i.). UMAV RNA in the samples was detected using the AgPath-ID One-Step RT-PCR Kit (Applied Biosystems, Carlsbad, CA, USA). The primer and probe sequences are provided in Table 1.

### 2.3. Virus Particle Electron Microscopy

The morphology of the virus particles was observed under electron microscopy. C6/36 cells were infected with the virus for three days, and the culture supernatant was centrifuged at 2000× *g* and 4 °C for 20 min to separate the supernatant and cell pellet.

For negative staining, cell culture supernatants were adsorbed onto 200-mesh carbon-coated copper grids for 3–5 min. Excess liquid was removed with filter paper. The grids were then stained with 2% phosphotungstic acid for 1–2 min, followed by removal of excess stain and air-drying at room temperature. The samples were observed under a transmission electron microscope (HITACHI HT7800/HT7700, Hitachi High-Tech Corporation, Tokyo, Japan) [21].

For ultrathin sections, cell pellets were fixed in TEM fixative, post-fixed with 1% osmium tetroxide in 0.1 M phosphate buffer (pH 7.4), and dehydrated through a graded ethanol series (30% to 100%) and acetone. Samples were infiltrated and embedded in EMBed 812 resin, polymerized at 60 °C for 48 h, and sectioned into 60–80 nm ultrathin slices using an ultramicrotome (Leica UC7). Sections were collected on 150-mesh formvar-coated copper grids, stained with 2% uranyl acetate and 2.6% lead citrate, and observed under a transmission electron microscope (HITACHI HT7800/HT7700, Hitachi High-Tech Corporation, Tokyo, Japan) [21].

### 2.4. Viral Titer Determination

The viral titer of isolates cultured in C6/36 cells was determined using a standard Plaque Formation Assay. Briefly, the virus was serially diluted ten-fold, and each dilution was inoculated in duplicate into six-well plates, followed by incubation at 28 °C for 1 h. After incubation, the cells were overlaid with 4 mL of 1.2% methylcellulose in 1640 medium supplemented with 2% fetal bovine serum. Following several days of culture, once distinct plaque formation became visible, the cells were stained with crystal violet solution, and the plaque-forming units (PFU) and viral titers were calculated [22].

### 2.5. Genome Sequence Determination

Viral RNA was extracted from the third-generation cell culture supernatant of a positive isolate using the CqEx RNA/DNA Nucleic Acid Extraction Kit (TIANLONG, Xi’an, China). A cDNA library was then constructed from the RNA using Protocol C of the VAHTS Universal V8 RNA-seq Library Prep Kit for Illumina (Vazyme, Nanjing, China). The resulting library was subjected to PE150 sequencing on an Illumina NovaSeq 6000 platform (Illumina, San Diego, CA, USA). The bioinformatic pipeline for viral genome reconstruction and validation was performed as follows: The raw sequencing reads were first processed using fastp with default parameters to remove adapter sequences and low-quality reads (Phred score < 15), generating high-quality clean data [23]. The clean reads were then de novo assembled into contigs using Megahit (v1.2.9) [24]. For viral sequence annotation, a two-step alignment strategy was adopted. First, contigs were aligned and annotated against a viral database using Diamond BLASTX (v2.1.16). Next, the putative viral sequences identified were extracted using the Meganizer tool (v6.25.10) and realigned against the non-redundant protein sequence database (nr database) for further annotation [25]. The final set of extracted viral sequences was mapped back to the original clean reads using the Burrows–Wheeler Aligner (BWA). Samtools (v1.22.1) was then used to count the aligned reads and calculate Reads Per Million (RPM) as a measure of viral abundance [26]. Sequences with an RPM value of less than 10 were considered false positives. The complete and reproducible Python (v3.10) script used in this study, has been uploaded on GitHub (https://github.com/wangruichen100/metaRNA, accessed on 8 November 2025) and is publicly available. The newly obtained genome sequences have been deposited in the GenBank database (https://www.ncbi.nlm.nih.gov/, accessed on 31 October 2025) under accession no. PX425590-9 and the GenBase database (https://ngdc.cncb.ac.cn/genbase/, accessed on 31 October 2025) under accession no. C_AA120364.1-73.1.

### 2.6. Sequence Alignment and Phylogenetic Analysis

All genetic sequences labeled as “Umatilla virus (UMAV)” were retrieved and downloaded from the National Center for Biotechnology Information (NCBI) GenBank database. Sequences lacking complete collection date, geographical location, host information, or those with incomplete genome sequences were excluded. The remaining sequences, covering different countries, time periods, and host origins, were combined with those newly obtained sequences in this study to form the final dataset, which included Yunnan orbivirus and Bluetongue virus as outgroups for comparative analysis. Multiple sequence alignment was performed using MAFFT v7.450. Phylogenetic trees of the ten gene segments were constructed by the Maximum Likelihood method in MEGA v11.0. Bootstrap values were calculated based on 1000 replicates [22]. Nucleotide and amino acid homology analyses were conducted using Geneious Prime (v11.0.15, 64 bit; Dotmatics, Auckland, New Zealand).

### 2.7. Transcriptome Sequencing Analysis

In this study, C6/36 cells cultured in T25 flasks were infected with NX23166 (MOI = 0.1). Mock-infected cells were processed in parallel as controls. Both infected and mock-infected cells were harvested at 24, 48, and 72 h.p.i., with three biological replicates per time point. Total RNA was extracted using the Total RNA Extractor (Trizol) kit (Sangon, Shanghai, China) and treated with DNase I to remove genomic DNA. RNA integrity, quality, and quantity were assessed via agarose gel electrophoresis, NanoPhotometer spectrophotometer (IMPLEN, Westlake Village, CA, USA), and Qubit 2.0 Fluorometer. Sequencing libraries were prepared from 1 μg RNA per sample using the VAHTSTM mRNA-seq V2 Library Prep Kit for Illumina, including mRNA purification, fragmentation, cDNA synthesis, end repair, adapter ligation, and size selection. Libraries were quantified and pooled for paired-end sequencing on an NovaSeq sequencers (Illumina, San Diego, CA, USA). Raw reads were quality-checked with FastQC (v0.11.2) and filtered using Trimmomatic (v0.36) to remove adapters and low-quality bases. Clean reads were aligned to the reference genome using HISAT2 (v2.0), with alignment quality assessed via RSeQC (v2.6.1), Qualimap (v2.2.1), and BEDTools (v2.26.0). Gene expression was quantified with StringTie (v1.3.3b) in Transcripts Per Million (TPM) units. Differential expression analysis was performed with DESeq2 (v1.12.4), applying thresholds of |FoldChange| ≥ 2 and *q*-value ≤ 0.001. Functional enrichment analysis of differentially expressed genes was conducted for KEGG pathways, with significance defined as *q*-value < 0.05 [27]. The transcriptome sequencing service was provided by Sangon Biotech (Shanghai, China) Co., Ltd.

## 3. Results

### 3.1. Viral Genome Sequencing and Assembly

**Figure 1:**
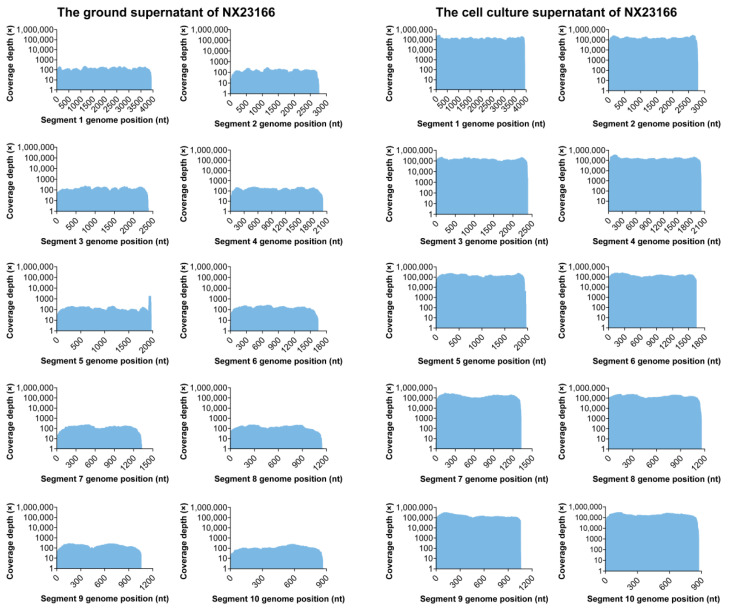
Comparison of next-generation sequencing depth for NX23166 between the ground supernatant and cell culture supernatant.

We performed cell culture inoculation on 185 sample pools and observed suspected CPEs only in pool NX23166, which originated from *Culex pipiens pallens* collected in Xiji County. Subsequent next-generation sequencing (NGS) and bioinformatics analysis of the second-passage cell culture supernatant identified UMAV, with no reads mapping to other mosquito-borne viruses. Furthermore, NGS analysis of the original 31 groups of *Culex pipiens pallens* samples revealed UMAV-related reads only in the original homogenate sample of NX23166, with no detection in the remaining samples. However, the sequencing data from this original sample showed incomplete viral genome coverage and low depth (Figure 1), with only 24,660 reads mapping to the UMAV genome. In contrast, sequencing of the cell culture supernatant yielded 21,982,308 UMAV-mapped reads, providing coverage across all ten segments, and the assembled viral genome segments ranged in size from 873 to 3928 nt (Table 2). These results indicate that UMAV was the only virus effectively isolated and amplified in cell culture from this sample pool.

### 3.2. Virus Isolation and Identification

**Figure 2:**
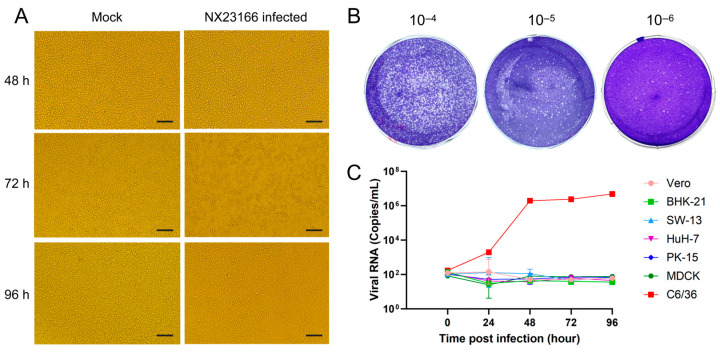
(**A**) Cytopathic effects in C6/36 cells infected with NX23166 or mock-infected at 48, 72 and 96 h.p.i. Scale bar: 100 μm. (**B**) Viral titer of the third-passage NX23166 virus. Plaque assay showing viral titer following serial 10-fold dilutions. (**C**) Growth kinetics of NX23166 in various cell lines, as determined by real-time quantitative polymerase chain reaction.

CPEs were continuously observed in C6/36 cells inoculated with the second-passage supernatant of NX23166. The CPEs became apparent at 48 h.p.i. With the extension of infection to 72 h.p.i., cell density further decreased, intercellular gaps widened, and partial cell rupture and death occurred. By 96 h.p.i., the CPEs had intensified significantly, characterized by large-scale cell rupture and death, a sharp decline in adherent cell density, and loss of monolayer integrity. Microscopic observation revealed extensive granular cellular debris deposited across the bottom of the culture plate (Figure 2A). The plaque assay revealed a virus titer of 2.7 × 10^8^ PFU/mL on 5 days post-inoculation of the fifth-generation virus (Figure 2B).

In this study, seven cell lines were selected for the infection experiments. The results showed that the NX23166 strain exhibited obvious CPEs only in C6/36 cells, appearing as early as 48 h.p.i. Viral growth kinetics analysis revealed that within 0–24 h.p.i., the virus replicated rather slowly; within 24–48 h.p.i., it entered the logarithmic growth phase; and after 48 h.p.i., the replication rate gradually slowed and stabilized. No viral replication was detected in the other six cell lines (Figure 2C).

### 3.3. Virus Electron Microscopy

**Figure 3:**
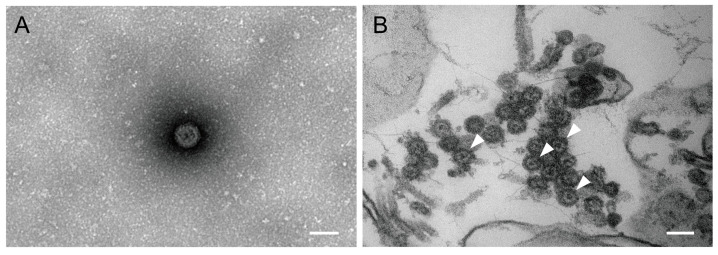
Electron micrographs of Umatilla virus (NX23166) particles. (**A**) Virus particles in the culture supernatant of infected C6/36 cells. (**B**) Virus particles within infected C6/36 cells. Arrows indicate representative virions. Scale bars, 100 nm.

Further morphological observation and analysis of the viral isolate were performed using negative staining and ultrathin sections combined with transmission electron microscopy. UMAV particles were observed in both the C6/36 supernatant and cells. The viral particles appeared spherical, lacked an envelope membrane, and displayed no obvious spikes or projections on the capsid surface, with a diameter of approximately 70 nm (Figure 3).

### 3.4. Phylogenetic Analysis

**Figure 4:**
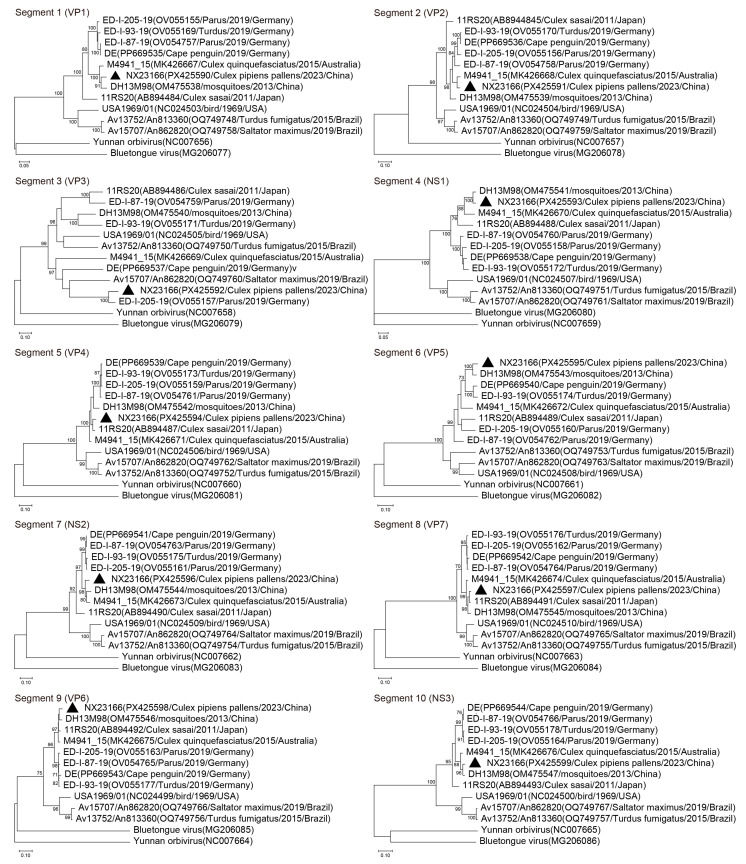
Phylogenetic analysis using Maximum Likelihood tree based on nucleotide sequences of all 10 segments of the UMAV strains. Gene sequence information includes “strain name (accession number/host/collection date/country)”. “▲” highlights the strain (NX23166) isolated in this study. Numbers associated with branches indicate percentages of 1000 bootstrap replicates supporting the existence of these branches. Branches with <70% bootstrap support have been collapsed. Yunnan orbivirus and Bluetongue virus as outgroups.

A dataset was constructed for analysis, comprising the newly sequenced NX23166 strain and 10 publicly available UMAV sequences with complete genomes from diverse countries, years, and hosts. Phylogenetic (Figure 4) and sequence homology (Table 3) analyses revealed the following: The VP1 gene of NX23166 strain clustered with those of the 2013 Yunnan (DH13M98) and 2015 Australian (M4941_15) isolates, with nucleotide (amino acid) identities of 94.87% (99.00%) and 95.06% (99.00%), respectively. The VP2 gene was most closely related to the M4941_15 strain, with nucleotide (amino acid) identity of 95.26% (99.67%). In contrast, the VP3 gene showed the highest identity to the 2019 German isolate (ED-I-205-19), with nucleotide (amino acid) identity of 87.75% (94.00%), while its identity to other strains was substantially lower (52.94–56.61% nucleotide; 47.38–51.49% amino acid). Finally, both the VP4 and VP7 genes clustered with the 2011 Japanese isolate (11RS20), with respective nucleotide (amino acid) identities of 95.95% (97.35%) and 98.01% (99.74%), respectively.

### 3.5. Transcriptomic Response to NX23166 (UMAV) Infection

**Figure 5:**
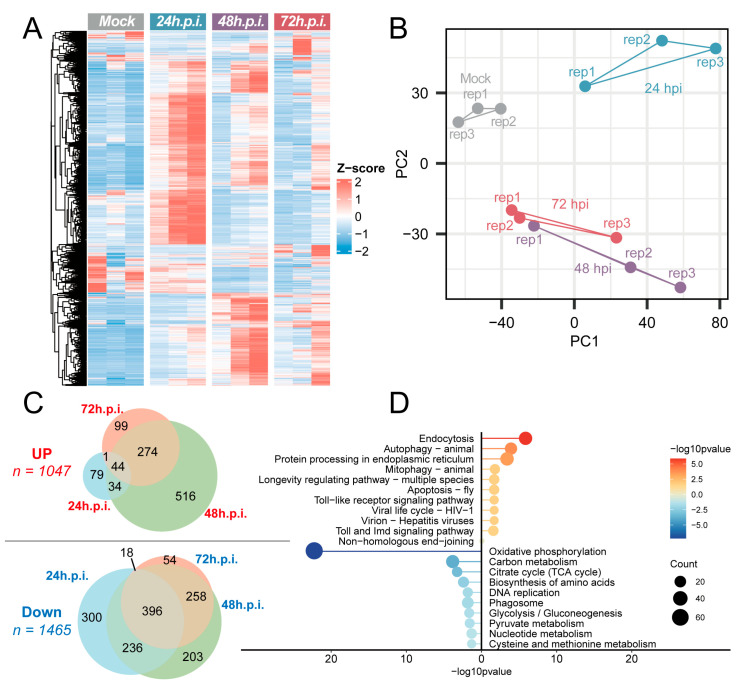
Transcriptomic profiling of C6/36 cells infected with Umatilla virus (NX23166). (**A**) Z-score heatmap of gene expression. Red indicates high expression, blue indicates low expression. (**B**) Principal component analysis plot. The mock control group, 24 h.p.i., 48 h.p.i., and 72 h.p.i. groups are represented in gray, blue, purple, and red, respectively. The labels rep1, rep2, and rep3 denote three biological replicates. (**C**) Venn diagrams of differentially expressed genes (DEGs). The Venn diagrams display the overlap of DEGs across different time points. The top and bottom diagrams illustrate the overlaps among upregulated and downregulated DEG sets, respectively. (**D**) KEGG pathway enrichment bubble plot. The plot displays the top 10 most significantly enriched KEGG pathways. Bubble size corresponds to the number of genes enriched in the pathway; bubble color indicates the gene set used for the enrichment analysis (red, analysis with the up-regulated gene set; blue, analysis with the down-regulated gene set); and the position on the x-axis represents the statistical significance of the enrichment (−log_10_ (*p*-value)).

Based on transcriptomic sequencing analysis of NX23166 (UMAV) infection, this study systematically elucidated the impact of viral infection on dynamic host gene expression changes and functional pathway regulation. After infecting C6/36 cells with NX23166 at an MOI of 0.1, infected cells were collected at three time points—24, 48, and 72 h post-infection (h.p.i.)—for transcriptomic sequencing analysis. The complete transcript abundance (TPM) for all genes across each sample is provided as supplementary data (Table S1). The results showed extensive perturbations in the transcriptional profiles (Figure 5A). The gene expression in the control group remained highly stable, with Z-scores concentrated near zero, while viral infection induced significant time-dependent changes in host gene expression. 

The comprehensive lists of up-regulated and down-regulated genes are detailed in Tables S2 and S3, respectively, while the full statistical results of the differential expression analyses at each infection time point are available in Table S4 (24 h.p.i.), Table S5 (48 h.p.i.), and Table S6 (72 h.p.i.). Principal component analysis (PCA) revealed that the transcriptional profiles at 48 h.p.i. and 72 h.p.i. were relatively close to each other (Figure 5B). Differential expression analysis, compared to the control group, identified 158, 834, and 418 up-regulated genes and 950, 1093, and 726 down-regulated genes at 24, 48, and 72 h.p.i., respectively. After removing redundant data, a total of 1047 time-dependent up-regulated genes and 1465 time-dependent down-regulated genes associated with infection were obtained (Figure 5C).

KEGG enrichment analysis of these time-dependent up-regulated and down-regulated genes revealed the top 10 up-regulated pathways, including: Endocytosis, Autophagy—animal, Protein processing in endoplasmic reticulum, Mitophagy—animal, Longevity regulating pathway—multiple species, Apoptosis—fly, Toll signaling pathway, Viral life cycle—HIV-1, Virion—Hepatitis viruses, and Toll and Imd signaling pathway (Table S7). The top 10 down-regulated pathways included: Oxidative phosphorylation, Carbon metabolism, Citrate cycle (TCA cycle), Biosynthesis of amino acids, DNA replication, Phagosome, Glycolysis/Gluconeogenesis, Pyruvate metabolism, Nucleotide metabolism, and Cysteine and methionine metabolism (Table S8). Among the pathways enriched for down-regulated genes, Oxidative phosphorylation was the most significantly enriched (Figure 5D).

## 4. Discussion

In a previous study, our research team detected the RNA of Tahyna virus (1 group), Japanese encephalitis virus (1 group), and Culex flavivirus (55 groups) in mosquitoes collected from the Ningxia Hui Autonomous Region in 2023 [19]. In the present study, the same mosquito samples were inoculated into two cell lines, and UMAV was successfully isolated from the *Culex pipiens pallens* pool NX23166, collected in Xiji County, using C6/36 cells. Subsequent NGS analysis of the viral isolation supernatant confirmed the presence of UMAV as the exclusive pathogen, with no reads mapping to other mosquito-borne viruses. This conclusion was further supported by NGS screening of the original 31 *Culex pipiens pallens* samples, which detected UMAV-related reads only in the NX23166 pool. Consistent with previous findings, UMAV has predominantly been isolated from *Culex* mosquitoes, suggesting that *Culex* species may play an important role in the transmission of UMAV and related viruses [3,11,16,17]. Moreover, the wide distribution of *Culex pipiens pallens*, a major mosquito species in northern China, indicates that UMAV has the potential to spread across a broader geographical area [28]. This isolation is the second reported isolation of UMAV in China since the Yunnan strain (YN2013) was identified in 2013, and its first discovery in Northwestern China.

NGS of the isolate yielded nearly complete sequences for all 10 genomic segments of the NX23166 strain. This strain exhibits typical characteristics of the genus *Orbivirus*. Its genome consists of 10 linear dsRNA segments, encoding 7 structural proteins and 3 non-structural proteins [9]. Morphological observation via transmission electron microscopy revealed that the viral particles have a typical spherical structure, approximately 70 nm in diameter, with a smooth surface and non-envelope [17]. These morphological features are highly consistent with those of previously reported UMAV strains. Its natural transmission cycle mainly involves avian hosts and mosquito vectors, facilitated by mosquito bites. However, no human infections have been reported [9].

According to the latest updates in viral genome databases, a total of 13 UMAV strains with relatively complete sequences are currently archived. Among them, 10 representative strains possess complete genome sequences of all 10 segments, primarily distributed across the following regions: (i) In Europe, four German isolates (OV054757, OV055169, OV055155, and PP669535) with complete genomes are documented [15,29]; (ii) In Asia, one Chinese Yunnan isolate (OM475538) and one Japanese isolate (AB894484) are available [3,17]; (iii) In the Americas, documented strains include the U.S. prototype strain (NC_024503) and two isolates from Brazil (OQ749748 and OQ749758) [9,30]; (iv) In Oceania, the Australian isolate (MK426667) has been documented as the representative strain [16]. This intercontinental distribution pattern not only reflects the broad adaptability of UMAV to diverse geographical environments, but also suggests its potential for long-distance dispersal through natural routes such as bird migration [31]. Emerging evidence indicates that the host range of UMAV is gradually expanding. German researchers, for the first time, isolated UMAV from the liver of a penguin that died in 2019. This finding confirms that the virus can infect marine birds and may exhibit hepatotropism, suggesting a potential risk of cross-species transmission [15].

Phylogenetic analysis revealed that the NX23166 strain is evolutionarily closely related to UMAV strains from Yunnan, Australia, and Germany, suggesting these strains share a common ancestor. The polymerase (Pol), subcore-shell (T2), and outercore (T13) proteins of orbiviruses are highly conserved, with >73%, >83%, and >73% amino acid identity, respectively, serving as the criteria for classification within the same species [32,33]. The NX23166 strain shares 89.10–99.00%, 94.75–99.67%, and 91.15–99.74% amino acid identity with other UMAV members in the Pol, T2, and T13 proteins, respectively. These values are well above the threshold required for virus species classification, confirming that NX23166 belongs to the UMAV species. In mosquito-borne orbiviruses, the OCP1 that determines the serotype is encoded by segment 3 (VP3) [3]. The NX23166 strain shows the highest nucleotide (amino acid) sequence identity of 87.75% (94.00%) to the German 2019 isolate ED-I-205-19 in this protein. In contrast, its VP3 (OCP1) gene identity with other isolates ranges from 47.38% to 51.49%. This suggests that NX23166 and ED-I-205-19 may belong to the same serotype, although further confirmation by serum neutralization tests is required.

In this study, we used transcriptomic sequencing data from NX23166 (UMAV)-infected C6/36 cells to reveal, for the first time, that this virus triggers highly dynamic host transcriptome reprogramming, and we identify several key up-regulated and down-regulated genes. It should be noted that, similar to studies on mosquito-borne viruses such as dengue virus and bluetongue virus [34,35], the C6/36 cell line used in this study is widely employed in virus–host interaction research due to its deficient RNA interference pathway [36]. This characteristic may enhance the sensitivity for observing the activation of immune pathways (such as the Toll signaling pathway and autophagy), while also providing a clearer perspective for revealing the direct regulatory mechanisms of viruses on fundamental host metabolic processes.

In recent years, increasing evidence has indicated that autophagy plays a crucial role in the replication and pathogenesis of animal viruses, suggesting that the modulation of autophagy may represent a novel therapeutic strategy against virus infection [37]. The up-regulation of autophagy-related gene expression detected in host cells after NX23166 infection indicates a close interaction between the virus and host autophagy activity. Furthermore, virus-induced mitophagy disrupts the signaling function of mitochondria in regulating the inflammation and immune responses, thereby contributing to the establishment of persistent viral infection [38]. This study observed up-regulated expression of the key genes in the mitophagy pathway, suggesting their potential involvement in the mechanism of viral persistence.

The Toll signaling pathway, a core component of the innate immune system, recognizes pathogen-associated molecular patterns (PAMPs) and damage-associated molecular patterns (DAMPs), thereby initiating immune responses [39]. This pathway demonstrates a dual role in various viral infections (such as SARS-CoV-2 and HCV): appropriate activation can promote immune protection, whereas excessive activation may trigger a cytokine storm and tissue damage [40]. The up-regulation of the host Toll signaling pathway observed after NX23166 infection indicates a successful activation of the innate immune defense response, suggesting that this response may be a key antiviral mechanism employed by the host to restrict viral replication and spread.

At the cellular metabolic level, oxidative phosphorylation (OXPHOS), as a central pathway of cellular energy metabolism, plays a crucial role in maintaining organismal homeostasis [41]. Previous studies have shown that various viruses can reprogram host cell metabolism by interfering with OXPHOS to promote their own replication and pathogenesis. For example, Zika virus (ZIKV) infection of neural stem cells forces a premature metabolic shift towards OXPHOS by activating p53 and inhibiting the mTOR pathway, leading to differentiation arrest and cell depletion [42]. In contrast, Hepatitis C virus (HCV) suppresses OXPHOS function by downregulating mitochondrial respiratory chain complex subunits (such as MT-ND1, MT-CO2), inducing a glycolytic dominance state similar to the Warburg effect, thereby providing metabolic precursors for viral proliferation and promoting liver disease progression [43]. Notably, this study also found that NX23166 infection significantly down-regulated the expression of genes related to the OXPHOS pathway. This finding suggests that the virus may disrupt the host cell’s energy supply through a similar metabolic reprogramming mechanism to support its own replication and survival.

## 5. Conclusions

This study successfully isolated and identified Umatilla virus (UMAV) from mosquito samples collected in the Ningxia region of China and characterized its fundamental genomic structure and transcriptomic features. These findings not only confirm the presence of UMAV in this area but also provide crucial data for understanding the genetic evolution of this virus. However, this study has certain limitations. For instance, the sample sources are relatively limited, and the research primarily focuses on genomic characterization. The biological properties and public health risks of the virus remain to be further evaluated. Nonetheless, this work lays an important molecular foundation for future investigations into the ecological distribution of UMAV, while also highlighting the significance of ongoing arbovirus surveillance.

## Figures and Tables

**Table 1:** Primer and probe sequences for detecting UMAV by RT-qPCR.

UMAV	The Primer and Probe Sequences (5′–3′)
Forward	TCCATGACTCTTGAGCCTGT
Reverse	TGTTTCAATCCTTGCACCGC
Probe	HEX-TGTCCGGATTCGTTGGCCCTCCA-BHQ2

Abbreviations: UMAV, Umatilla virus; RT-qPCR, real-time quantitative polymerase chain reaction.

**Table 2:** Next-generation sequencing of RNA from the ground supernatant and cell culture supernatant of NX23166.

Segment	Genes	Next-Generation Sequencing				Putative Protein
		The Ground Supernatant (bp)	Reads	The Cell Culture Supernatant (bp)	Reads	
1	VP1	3912	4503	3928	4,066,811	RNA dependent RNA Polymerase (Pol)
2	VP2	2764	3400	2780	3,223,049	Major subcore protein (T2)
3	VP3	2381	2735	2381	2,615,661	Outer capsid protein (OCP1)
4	NS1	1999	2667	2018	2,477,484	Tubule protein (TuP)
5	VP4	1978	3865	1994	2,244,156	Minor core protein-capping enzyme (Cap)
6	VP5	1639	2161	1639	1,819,865	Outer capsid protein (OCP2)
7	NS2	1308	1555	1324	1,623,891	Viral inclusion body protein (ViP)
8	VP7	1156	1328	1157	1,379,620	Major core-surface protein (T13)
9	VP6	1052	1588	1072	1,284,522	Minor core protein-helicase enzyme (Hel)
10	NS3	867	858	873	1,247,249	Virus release protein (VRP)

**Table 3:** Nucleotide and amino acid homology analysis of NX23166 and other UMAV strains.

**Virus Strains**	**Nucleotide Sequence Identity (%)**									
NX23166	Seg1	Seg2	Seg3	Seg4	Seg5	Seg6	Seg7	Seg8	Seg9	Seg10
DH13M98	94.87	90.11	55.67	97.16	90.40	94.46	93.41	95.67	96.64	97.11
M4941_15	95.06	95.26	53.28	89.36	94.67	84.67	93.88	95.41	96.93	95.36
ED-I-87-19	88.12	90.34	52.94	84.67	90.34	84.91	91.98	89.90	91.75	89.11
ED-I-93-19	88.30	89.72	53.80	85.23	90.41	86.58	91.25	89.74	91.94	88.89
ED-I-205-19	88.09	89.67	87.75	84.36	90.34	84.90	91.90	89.61	91.55	88.75
DE	88.20	89.40	56.21	83.73	89.75	86.70	91.76	88.76	91.26	87.68
11RS20	85.65	87.51	54.46	86.38	95.95	82.76	85.88	98.01	96.64	87.98
Av13752/An813360	75.26	78.30	53.52	71.70	76.00	74.72	72.02	80.96	73.03	66.91
Av15707/An862820	75.26	78.24	56.61	72.40	76.85	76.66	71.61	81.80	73.64	66.50
USA1969/01	76.38	78.23	53.73	71.96	72.04	75.18	72.07	81.89	74.38	67.89
**Virus Strains**	**Amino Acid Sequence Identity (%)**									
NX23166	VP1	VP2	VP3	NS1	VP4	VP5	NS2	VP7	VP6	NS3
DH13M98	99.00	99.24	49.24	98.95	97.04	99.26	99.29	99.48	96.25	98.25
M4941_15	99.00	99.67	47.44	98.50	97.73	96.67	99.53	99.74	97.12	98.60
ED-I-87-19	98.16	99.24	47.38	96.68	97.02	97.41	99.05	97.10	91.64	97.53
ED-I-93-19	98.08	98.80	48.85	96.70	96.24	97.60	99.05	97.13	91.64	97.20
ED-I-205-19	98.08	99.01	94.00	96.36	96.72	96.11	99.05	97.09	91.35	96.72
DE	98.23	98.87	51.49	96.49	96.17	97.23	99.01	96.77	90.96	97.98
11RS20	97.55	98.92	48.03	96.71	97.35	94.82	94.34	99.74	95.68	97.55
Av13752/An813360	89.33	95.35	49.15	78.88	86.35	86.43	79.62	91.83	64.62	66.79
Av15707/An862820	89.10	94.75	50.89	80.00	86.18	88.17	79.42	92.16	65.99	67.44
USA1969/01	89.50	95.12	47.79	79.64	80.18	89.09	80.19	91.15	66.28	69.58

NOTE: VP1 (Pol); VP2 (T2); VP3 (OCP1); NS1 (TuP); VP4 (Cap);VP5 (OCP2); NS2 (ViP); VP7 (T13); VP6 (Hel); NS3 (VRP). The underlined regions indicate the highest nucleotide and amino acid identity in segment 3.

## Data Availability

The data supporting the findings of this study are freely available in the GenBank (https://www.ncbi.nlm.nih.gov/, accessed on 31 October 2025) and GenBase (https://ngdc.cncb.ac.cn/genbase/, accessed on 31 October 2025) databases, under reference numbers PX425590-9 and C_AA120364.1-73.1, respectively.

## Supplementary Materials

The following supporting information (Transcriptomics analysis results) can be downloaded at: https://www.mdpi.com/article/10.3390/microorganisms13122717/s1. Table S1. Transcript abundance (TPM) of genes across different samples; Table S2. List of upregulated genes; Table S3. List of downregulated genes; Table S4. Differential gene expression analysis at 24 h post-infection (24hpi); Table S5. Differential gene expression analysis at 48 h post-infection (48hpi); Table S6. Differential gene expression analysis at 72 h post-infection (72hpi); Table S7. KEGG pathway enrichment analysis of upregulated genes in Aedes albopictus; Table S8. KEGG pathway enrichment analysis of downregulated genes in Aedes albopictus.

## Abbreviations

The following abbreviations are used in this manuscript:

UMAV	Umatilla virus
dsRNA	double-stranded RNA
NGS	next-generation sequencing
RT-qPCR	real-time quantitative polymerase chain reaction
CPE	cytopathic effect
PFU	plaque-forming unit
h.p.i.	hours post-infection
PCA	Principal component analysis
